# Estimation of population parameters using sample extremes from nonconstant sample sizes

**DOI:** 10.1371/journal.pone.0280561

**Published:** 2023-01-20

**Authors:** Tiffany N. Kolba, Alexander Bruno

**Affiliations:** Department of Mathematics and Statistics, Valparaiso University, Valparaiso, IN, United States of America; Dartmouth College Geisel School of Medicine, UNITED STATES

## Abstract

We examine the accuracy and precision of parameter estimates for both the exponential and normal distributions when using only a collection of sample extremes. That is, we consider a collection of random variables, where each of the random variables is either the minimum or maximum of a sample of *n*_*j*_ independent, identically distributed random variables drawn from a normal or exponential distribution with unknown parameters. Previous work derived estimators for the population parameters assuming the *n*_*j*_ sample sizes are constant. Since sample sizes are often not constant in applications, we derive new unbiased estimators that take into account the varying sample sizes. We also perform simulations to assess how the previously derived estimators perform when the constant sample size is simply replaced with the average sample size. We explore how varying the mean, standard deviation, and probability distribution of the sample sizes affects the estimation error. Overall, our results demonstrate that using the average sample size in place of the constant sample size still results in reliable estimates for the population parameters, especially when the average sample size is large. Our estimation framework is applied to a biological example involving plant pollination.

## 1 Introduction

We consider a data set where each observation is the minimum or maximum of a sample of independent, identically distributed (iid) random variables drawn from a population with unknown parameters. That is, our data consists of $W_{j}=\;\text{min}\;{\left\{X_{ij}\right\}}_{i=1}^{n_{j}}$ or $Y_{j}=\;\text{max}\;{\left\{X_{ij}\right\}}_{i=1}^{n_{j}}$ for *j* = 1, …, *m*. Here *m* represents the number of known sample extremes, while *n*_*j*_ represents the sample size of the *j*th sample over which the minimum or maximum is computed. While the *X*_*ij*_ are iid draws from a population with unknown parameters, the *X*_*ij*_ values themselves are not directly observable. Rather, we consider the case where it is only possible to measure the minimum or maximum of each sample. We seek to use the minimum and maximum values in order to estimate the unknown population parameters.

In [1], estimators for the exponential and normal distributions were derived assuming the *n*_*j*_ sample sizes are constant. Those two population distributions were considered since they arise frequently in applications and since they allow for explicit derivations. However, constant sample sizes are unrealistic to occur in applications. For example, [2, 3] examined the fertilization process in the flowering plant *Arabidopsis thaliana*. Their experiments begin by placing pollen on the stigma of the plant; they then wait a certain time period, kill the plant, and image it in order to view the fertilization progress. In the images, the researchers are able to view the pollen tubes, which grow down from the stigma towards the plant ovules. When a pollen tube reaches an ovule, it can fertilize it, which will then develop into a seed. The average fertilization speed is of biological interest and can be calculated by dividing the average pollen tube length by the amount of time lapsed since the pollen were placed on the stigma. However, due to the high density of pollen tubes, it is only feasible for the researchers to measure the *longest* pollen tube within each plant, and yet they wish to use these longest measurements to estimate the overall average length. Hence, their data fits our framework of estimating a population parameter with sample extremes. The *n*_*j*_ sample sizes in this application represent the number of pollen tubes within the *j*th plant. It is not practical for researchers to place the same exact number of pollen grains on each plant, and hence the *n*_*j*_ values will naturally vary. While [1] estimated the average pollen tube length by assuming a constant sample size for the number of pollen tubes within each plant, we seek to improve the estimate by deriving a new estimator for the population mean that allows for the samples sizes themselves to be random variables.

In Section 2, which focuses on the case where the population is exponentially distributed, we derive new estimators for the population mean under increasingly more realistic assumptions for the *n*_*j*_ sample sizes. We also derive the variances of our new estimators, and consider cases where the *n*_*j*_ sample sizes follow either a uniform or Poisson distribution. We compare the accuracy and precision of our new estimators to the original estimator computed in [1] under the constant sample size assumption. When the population is normally distributed, it is not feasible to analytically derive unbiased estimators for the population parameters using sample extremes with nonconstant sample sizes. Hence, in Section 3, we focus solely on analyzing the performance of the original estimators from [1] for the normal distribution when the constant sample size assumption is violated and the average sample size is instead used. In Section 4, we compare our methodology to that of maximum likelihood estimation. Lastly, in Section 5, we apply our results to the plant pollination example.

## 2 Estimator for exponential distribution

In this section we assume that $X_{ij}\overset{\text{iid}}{∼}Exp\left(\beta \right)$ where *β* is the unknown population mean. We set $W_{j}=\;\text{min}\;{\left\{X_{ij}\right\}}_{i=1}^{n_{j}}$ and $Y_{j}=\;\text{max}\;{\left\{X_{ij}\right\}}_{i=1}^{n_{j}}$ for *j* = 1, …, *m*.

### 2.1 Sample sizes *n*_*j*_ are all equal to a known value *n*

In [1], it was shown that in the case of constant sample size *n*, the estimator

$$\begin{array}{c} {\hat{\beta }}_{1}=\frac{1}{mH_{n}}\sum_{j=1}^{m}Y_{j}=\frac{\bar{Y}}{H_{n}} \end{array}$$ (1)

is an unbiased estimator for *β* with

$$\begin{array}{c} \text{var}\left({\hat{\beta }}_{1}\right)=\frac{\beta^{2}G_{n}}{mH_{n}^{2}} \end{array}$$ (2)

where

$$H_{n}=\sum_{i=1}^{n}\frac{1}{i},\;G_{n}=\sum_{i=1}^{n}\frac{1}{i^{2}}.$$

While the prior work did not consider the case of a sample of sample minima, it follows directly from properties of the exponential distribution [4] and the methods in [1] that the estimator

$$\begin{array}{c} {\tilde{\beta }}_{1}=\frac{n}{m}\sum_{j=1}^{m}W_{j}=n\bar{W} \end{array}$$ (3)

is also an unbiased estimator for *β* with

$$\begin{array}{c} \text{var}\left({\tilde{\beta }}_{1}\right)=\frac{\beta^{2}}{m}. \end{array}$$ (4)

While there exists an unbiased estimator using either sample maxima or sample minima, we observe from Eqs (2) and (4) that the variance using sample maxima is strictly smaller with $\text{var}({\hat{\beta }}_{1})\to 0$ at rate proportional to $\frac{1}{{\left(\text{log}\;n\right)}^{2}}$ as *n* → ∞, while $\text{var}({\tilde{\beta }}_{1})$ is constant with respect to *n*. Hence, more precise estimates for the exponential distribution can be obtained from using maximum values compared to using minimum values.

### 2.2 Sample sizes *n*_*j*_ are unequal, but are all known values

As mentioned in Section 1, it is unrealistic for the sample sizes to be equal in applications. Hence, we begin to improve the estimation framework by allowing the sample sizes to vary, but with the restriction that the sample sizes are known values. It follows directly from properties of the exponential distribution [4] and the methods in [1] that the estimator

$$\begin{array}{c} {\hat{\beta }}_{2}=\frac{1}{m}\sum_{j=1}^{m}\frac{Y_{j}}{H_{n_{j}}} \end{array}$$ (5)

with variance

$$\begin{array}{c} \text{var}\left({\hat{\beta }}_{2}\right)=\frac{\beta^{2}}{m^{2}}\sum_{j=1}^{m}\frac{G_{n_{j}}}{H_{n_{j}}^{2}} \end{array}$$ (6)

and the estimator

$$\begin{array}{c} {\tilde{\beta }}_{2}=\frac{1}{m}\sum_{j=1}^{m}n_{j}W_{j} \end{array}$$ (7)

with variance

$$\begin{array}{c} \text{var}\left({\tilde{\beta }}_{2}\right)=\frac{\beta^{2}}{m} \end{array}$$ (8)

are unbiased for estimating the population mean *β*. The estimators introduced in Eqs (5) and (7) reduce to the previous estimators given in Eqs (1) and (3) when all the *n*_*j*_ are equal. Hence, these estimators are simply generalizations that allow them to be utilized in a broader range of applications. Yet, in some applications, not only do the sample sizes vary, but the sample sizes are also unknown. For example, in the plant pollination application described in Section 1, hundreds of pollen grains are placed on each plant and it is not possible for the researchers to count the exact numbers. Hence, the sample sizes are unknown, but the researchers do have an idea of the probability distribution of the sample sizes. In the following subsection, we further improve the estimation framework by considering the case where the sample sizes are themselves random variables.

### 2.3 Sample sizes *n*_*j*_ are random with known distribution

We now suppose that the *n*_*j*_ sample sizes are iid random variables. We assume that the *n*_*j*_ values are unknown, but come from some known probability distribution, such as the uniform or Poisson distribution. It immediately follows from the proof of Theorem 1 in [1] that

$$E\left[Y_{j}|n_{j}\right]=\beta H_{n_{j}}\;\text{and}\;\text{var}\left(Y_{j}|n_{j}\right)=\beta^{2}G_{n_{j}}.$$

By properties of conditional expectation and conditional variance [4],

$$E\left[Y_{j}\right]=E\left[E\left[Y_{j}|n_{j}\right]\right]=E\left[\beta H_{n_{j}}\right]=\beta \cdot E\left[H_{n_{j}}\right]$$

and

$$\begin{array}{cc} \text{var}(Y_{j}) & =\text{var}\left(E\left[Y_{j}|n_{j}\right]\right)+E\left[\text{var}\left(Y_{j}|n_{j}\right)\right]=\text{var}\left(\beta H_{n_{j}}\right)+E\left[\beta^{2}G_{n_{j}}\right]\\ & =\beta^{2}\left(\text{var}\left(H_{n_{j}}\right)+E\left[G_{n_{j}}^{2}\right]\right). \end{array}$$

Thus, the estimator

$$\begin{array}{c} {\hat{\beta }}_{3}=\frac{1}{m}\sum_{j=1}^{m}\frac{Y_{j}}{E[H_{n_{j}}]}=\frac{\bar{Y}}{E[H_{n_{j}}]} \end{array}$$ (9)

with

$$\begin{array}{c} \text{var}\left({\hat{\beta }}_{3}\right)=\frac{\beta^{2}}{m}\frac{\left(\text{var}\left(H_{n_{j}}\right)+E\left[G_{n_{j}}\right]\right)}{E{\left[H_{n_{j}}\right]}^{2}} \end{array}$$ (10)

is unbiased for estimating *β*. Likewise,

$$E\left[W_{j}\right]=E\left[E\left[W_{j}|n_{j}\right]\right]=E[\frac{\beta }{n_{j}}]=\beta \cdot E[\frac{1}{n_{j}}]$$

and

$$\begin{array}{cc} \text{var}(W_{j}) & =\text{var}\left(E\left[W_{j}|n_{j}\right]\right)+E\left[\text{var}\left(W_{j}|n_{j}\right)\right]=\text{var}(\frac{\beta }{n_{j}})+E[\frac{\beta^{2}}{n_{j}^{2}}]\\ & =\beta^{2}(\text{var}(\frac{1}{n_{j}})+E[\frac{1}{n_{j}^{2}}]). \end{array}$$

Hence, the estimator

$$\begin{array}{c} {\tilde{\beta }}_{3}=\frac{1}{m}\sum_{j=1}^{m}\frac{W_{j}}{E[\frac{1}{n_{j}}]}=\frac{\bar{W}}{E[\frac{1}{n_{j}}]} \end{array}$$ (11)

with

$$\begin{array}{c} \text{var}\left({\tilde{\beta }}_{3}\right)=\frac{\beta^{2}}{m}\frac{\left(\text{var}(\frac{1}{n_{j}})+E[\frac{1}{n_{j}^{2}}]\right)}{{\left(E[\frac{1}{n_{j}}]\right)}^{2}}=\frac{\beta^{2}}{m}(2\cdot \frac{E[\frac{1}{n_{j}^{2}}]}{{\left(E[\frac{1}{n_{j}}]\right)}^{2}}-1) \end{array}$$ (12)

is also unbiased for estimating *β*.

We observe from comparing Eqs (10) and (12) with Eqs (2) and (4), respectively, that the variance of the estimators is higher when the sample sizes are random variables compared to when the sample sizes are constant. This relationship is unsurprising since it is intuitive that higher variation in the sample sizes will result in higher variation in the estimation of the population mean.

In order to actually calculate the estimators ${\hat{\beta }}_{3}$ and ${\tilde{\beta }}_{3}$, the values of $E[H_{n_{j}}]$ and $E[\frac{1}{n_{j}}]$ must first be computed. These expected values can be computed exactly if the probability distribution of the *n*_*j*_ is known. For example, if $n_{j}\overset{\text{iid}}{∼}\text{Uniform}\{a,\cdots ,b\}$ with known values for *a* and *b*, then $E\left[n_{j}\right]=\frac{a+b}{2}$,

$$E\left[H_{n_{j}}\right]=\frac{1}{b-a+1}\sum_{n=a}^{b}\sum_{i=1}^{n}\frac{1}{i},$$

and

$$E[\frac{1}{n_{j}}]=\frac{1}{b-a+1}\sum_{n=a}^{b}\frac{1}{n}.$$

Or if the *n*_*j*_ follow a Poisson distribution (shifted to start at 1), i.e. $n_{j}-1\overset{\text{iid}}{∼}\text{Poisson}(\lambda )$ with known λ, then *E*[*n*_*j*_] = λ + 1,

$$E\left[H_{n_{j}}\right]=e^{-\lambda }\sum_{n=0}^{\infty }\frac{\lambda^{n}}{n!}\sum_{i=1}^{n+1}\frac{1}{i},$$

and

$$E[\frac{1}{n_{j}}]=e^{-\lambda }\sum_{n=0}^{\infty }\frac{\lambda^{n}}{(n+1)!}=\frac{1}{\lambda }\left(1-e^{-\lambda }\right).$$

### 2.4 Sample sizes *n*_*j*_ are random with only known mean

If the full probability distribution of the *n*_*j*_ is not known, but the average sample size, *E*[*n*_*j*_], is known, it would be reasonable to approximate $E[H_{n_{j}}]$ and $E[\frac{1}{n_{j}}]$ with $H_{E[n_{j}]}$ and $\frac{1}{E[n_{j}]}$, respectively. Using these approximations yields the following estimators:

$$\begin{array}{c} {\hat{\beta }}_{4}=\frac{1}{m}\sum_{j=1}^{m}\frac{Y_{j}}{H_{E[n_{j}]}}=\frac{\bar{Y}}{H_{E[n_{j}]}} \end{array}$$ (13)

and

$$\begin{array}{c} {\tilde{\beta }}_{4}=\frac{1}{m}\sum_{j=1}^{m}\frac{W_{j}}{\frac{1}{E[n_{j}]}}=E\left[n_{j}\right]\cdot \bar{W}. \end{array}$$ (14)

Since $H_{n_{j}}=\sum_{i=1}^{n_{j}}\frac{1}{i}$ is a concave function of *n*_*j*_, $E\left[H_{n_{j}}\right]<H_{E[n_{j}]}$ by Jensen’s inequality [5]. Hence, ${\hat{\beta }}_{4}$ is a biased estimator of *β* that tends to *underestimate* the true value of *β*. Conversely, since $\frac{1}{n_{j}}$ is a convex function of *n*_*j*_, $E\left[\frac{1}{n_{j}}\right]>\frac{1}{E[n_{j}]}$, which makes ${\tilde{\beta }}_{4}$ a biased estimator that tends to *overestimate* the true value of *β*.

Note that ${\hat{\beta }}_{4}$ and ${\tilde{\beta }}_{4}$ are equivalent to using the original estimators ${\hat{\beta }}_{1}$ and ${\tilde{\beta }}_{1}$ and replacing the constant sample size *n* with *E*[*n*_*j*_]. In the next subsection, we compare the performance of the various estimators.

### 2.5 Comparison of estimators

The estimators ${\hat{\beta }}_{2}$, ${\hat{\beta }}_{3}$, and ${\hat{\beta }}_{4}$ based upon a sample of sample maxima all reduce to the estimator ${\hat{\beta }}_{1}$ when the sample sizes *n*_*j*_ are all equal to a constant value *n*. Likewise, the estimators ${\tilde{\beta }}_{2}$, ${\tilde{\beta }}_{3}$, and ${\tilde{\beta }}_{4}$ based upon a sample of sample minima all reduce to the estimator ${\tilde{\beta }}_{1}$ in the case of known equal sample sizes. As mentioned previously, the estimators ${\hat{\beta }}_{2}$ and ${\tilde{\beta }}_{2}$ allow for unequal sample sizes, but still require that the sample sizes are known values. On the other hand, the estimators ${\hat{\beta }}_{3}$, ${\tilde{\beta }}_{3}$, ${\hat{\beta }}_{4}$, and ${\tilde{\beta }}_{4}$ allow for the sample sizes to be unknown iid random variables, which is the most realistic scenario to occur in applications.

Although ${\hat{\beta }}_{3}$, ${\tilde{\beta }}_{3}$, ${\hat{\beta }}_{4}$, and ${\tilde{\beta }}_{4}$ all allow for random sample sizes, ${\hat{\beta }}_{3}$ and ${\tilde{\beta }}_{3}$ require that the full probability distribution of the sample sizes is known, while ${\hat{\beta }}_{4}$ and ${\tilde{\beta }}_{4}$ only require the average sample size to be known. Thus, the estimators ${\hat{\beta }}_{4}$ and ${\tilde{\beta }}_{4}$, which are equivalent to the original estimators ${\hat{\beta }}_{1}$ and ${\tilde{\beta }}_{1}$ when the constant sample size is simply replaced with the average sample size, have the advantage of applying in the broadest range of circumstances. Yet, they have the disadvantage of being the only biased estimators presented here.

**Fig 1:**
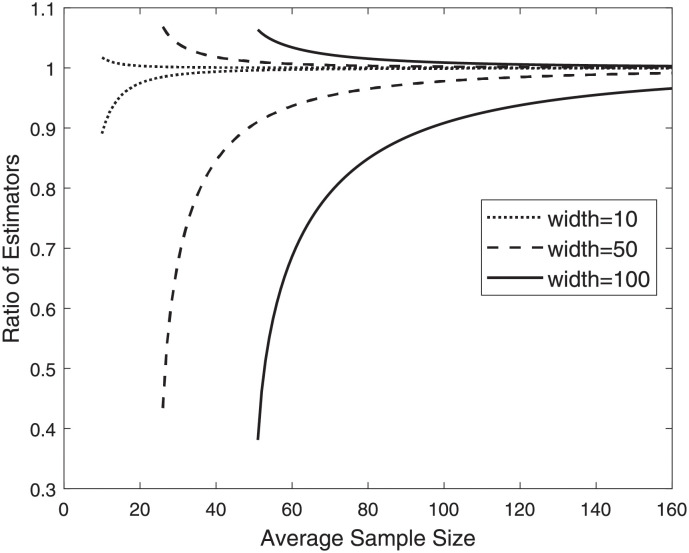
Ratio of ${\hat{\beta }}_{3}$ to ${\hat{\beta }}_{4}$ (curves above one) and ratio of ${\tilde{\beta }}_{3}$ to ${\tilde{\beta }}_{4}$ (curves below one) when the samples sizes are uniformly distributed with varying average and width of uniform interval.

Fig 1 illustrates the ratio between ${\hat{\beta }}_{3}$ and ${\hat{\beta }}_{4}$, which is equal to the ratio between $1/E[H_{n_{j}}]$ and $1/H_{E\left[n_{j}\right]}$, as well as the ratio between ${\tilde{\beta }}_{3}$ and ${\tilde{\beta }}_{4}$, which is equal to the ratio between $1/E[\frac{1}{n_{j}}]$ and *E*[*n*_*j*_], in the case where the sample sizes follow a uniform distribution. We observe in Fig 1 that the ratio when using sample maxima (${\hat{\beta }}_{3}/{\hat{\beta }}_{4}$) is always greater than one since ${\hat{\beta }}_{4}$ tends to underestimate the true population mean. In contrast, the ratio when using sample minima (${\tilde{\beta }}_{3}/{\tilde{\beta }}_{4}$) is always less than one since ${\tilde{\beta }}_{4}$ tends to overestimate the true population mean. In either case, the ratio between the two estimators is further from one when the width of the uniform distribution is larger since a wider interval results in more variability in the sample sizes. However, the ratio of the two estimators converges to one as the average sample size *E*[*n*_*j*_] increases. The convergence is faster when using sample maxima compared to when using sample minima. Similar patterns can be observed when the sample sizes follow other probability distributions, such as the Poisson distribution. Overall, Fig 1 illustrates that although ${\hat{\beta }}_{4}$ and ${\tilde{\beta }}_{4}$, which simply use the average sample size, are biased, they can still result in good estimates, especially when the average sample size is large.

## 3 Estimators for normal distribution

We now consider the case where $X_{ij}\overset{\text{iid}}{∼}$ Normal with unknown mean *μ* and unknown variance *σ*^2^. We again set $W_{j}=\;\text{min}\;{\left\{X_{ij}\right\}}_{i=1}^{n_{j}}$ and $Y_{j}=\;\text{max}\;{\left\{X_{ij}\right\}}_{i=1}^{n_{j}}$ for *j* = 1, …, *m*.

### 3.1 Sample sizes *n*_*j*_ are all equal to a known value *n*

The estimators given in [1] when using a sample of maximum values and assuming a constant sample size *n* are

$$\begin{array}{c} \hat{\mu }=\bar{Y}-\frac{k_{n}}{\sqrt{c_{n}}}S_{Y},\;{\hat{\sigma }}^{2}=\frac{S_{Y}^{2}}{c_{n}}. \end{array}$$ (15)

Here $\bar{Y}$ and $S_{Y}^{2}$ denote the sample mean and sample variance, respectively, of the *Y*_*j*_’s, while *k*_*n*_ denotes the mean and *c*_*n*_ denotes the variance of the maximum of *n* iid Normal(0, 1) random variables. It was shown that ${\hat{\sigma }}^{2}$ is an unbiased estimator for *σ*^2^, but that $E(\hat{\mu })>\mu$ with $E(\hat{\mu })\to \mu$ as *m* → ∞.

Although [1] did not consider the case of a sample of minimum values, we can define the analogous estimators

$$\begin{array}{c} \tilde{\mu }=\bar{W}+\frac{k_{n}}{\sqrt{c_{n}}}S_{W},\;{\tilde{\sigma }}^{2}=\frac{S_{W}^{2}}{c_{n}}, \end{array}$$ (16)

where now $\bar{W}$ and $S_{W}^{2}$ denote the sample mean and sample variance, respectively, of the *W*_*j*_’s. The constants *k*_*n*_ and *c*_*n*_ appear in both sets of estimators since the mean of the minimum of *n* iid Normal(0, 1) random variables is equal to the negative of the mean of the maximum of *n* iid Normal(0, 1) random variables, while the variance is the same in both cases. The values for *k*_*n*_ and *c*_*n*_ can be approximated either analytically or through simulations [6].

Due to the symmetry of the normal distribution, the estimators using minimum values will have equivalent performance to the estimators using maximum values. More specifically, it follows from symmetry that ${\tilde{\sigma }}^{2}$ is also an unbiased estimator for *σ*^2^ and that $E(\tilde{\mu })<\mu$ with $E(\tilde{\mu })\to \mu$ as *m* → ∞. This equivalent performance using either sample maxima or sample minima is in stark contrast to the case when the population is exponentially distributed. For an exponential population, it was shown in Section 2 that it is more advantageous to estimate the population parameters using a sample of maximum values due to the significantly smaller variance of the estimators in the maxima setting compared to the minima setting.

### 3.2 Sample sizes *n*_*j*_ are random with known mean

Due to the complexity of the normal probability density function, it is not feasible to derive unbiased estimators for *μ* and *σ*^2^ in the case where the sample sizes are unequal known values or are random variables with a known distribution. However, if the *n*_*j*_ values are iid random variables with known average sample size, *E*[*n*_*j*_], it is reasonable to replace the *k*_*n*_ and *c*_*n*_ values in Eqs (15) and (16) with $k_{E[n_{j}]}$ and $c_{E[n_{j}]}$, respectively.

We evaluate the performance of the estimators when the constant sample size *n* is replaced with the average sample size *E*[*n*_*j*_] through simulations. Although the true population mean *μ* and true population variance *σ*^2^ are unknown, we set them equal to 0 and 1, respectively, for our simulations so that we can explicitly compare in our simulations how close $\hat{\mu }$ and ${\hat{\sigma }}^{2}$ are to the true values. True values other than 0 or 1 would shift and rescale the distribution, but would not affect the relative performance of the estimators. Our simulations focus on $\hat{\mu }$ and ${\hat{\sigma }}^{2}$, which are based on sample maxima, but due to the symmetry of the normal distribution, $\tilde{\mu }$ and ${\tilde{\sigma }}^{2}$, which are based on sample minima, would have analogous performance.

**Fig 2:**
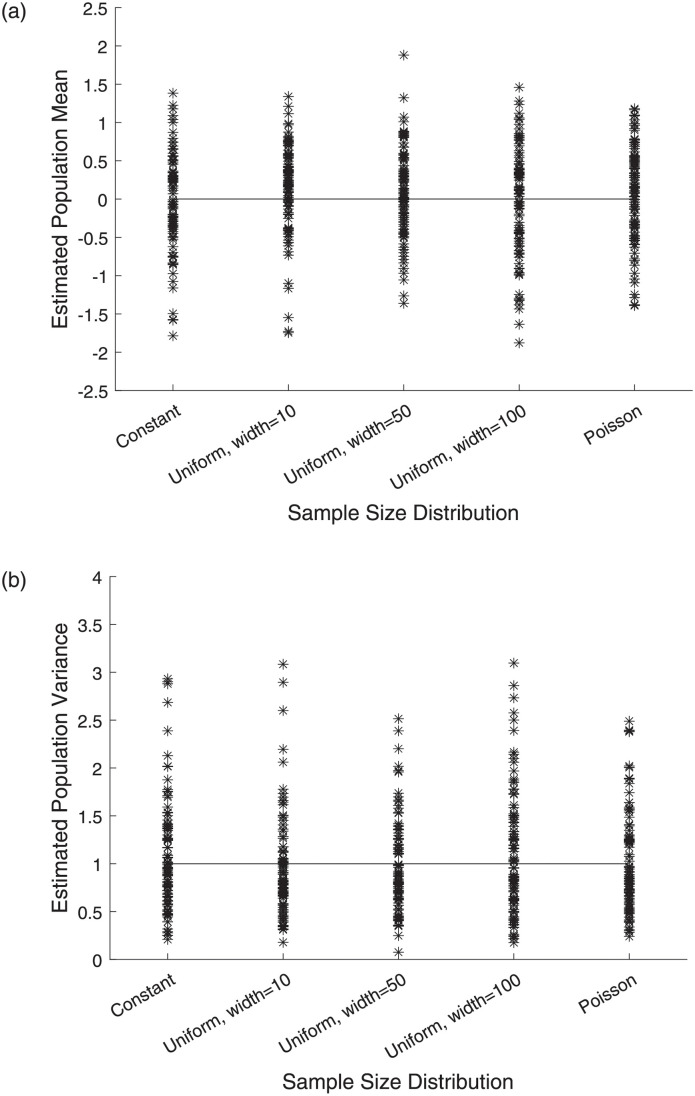
Estimates of the population mean (top) and population variance (bottom) from 100 simulations using *m* = 10 sample maxima. The maxima are computed over samples with average size *E*[*n*_*j*_] = 100 for various distributions of the *n*_*j*_.

Fig 2 compares the performance of both $\hat{\mu }$ (top) and ${\hat{\sigma }}^{2}$ (bottom) when the distribution of the sample sizes *n*_*j*_ is constant, uniform with width 10, uniform with width 50, uniform with width 100, or Poisson. In all cases, *m* = 10 sample maxima are used, where each maximum is computed over a sample with average size *E*[*n*_*j*_] = 100. We observe from Fig 2 that the accuracy and precision of the estimators is essentially identical regardless of whether the sample sizes are constant or randomly distributed. Increasing the variability of the sample sizes through a larger width of the uniform interval does not have a noticeable effect. While changing the values of *m* and *E*[*n*_*j*_] does change the variability of the simulated distributions, it does not alter the relative performance across the various distributions of *n*_*j*_. Thus, we conclude that when estimating the parameters of the Normal distribution using either sample maxima or sample minima, the constant sample size can be replaced with the average sample size with no significant change in the accuracy and precision of the estimation.

## 4 Comparison to maximum likelihood estimation

Suppose *X*_*ij*_ are drawn iid from an arbitrary population with probability density function *f*(*x*|Θ) and cumulative distribution function *F*(*x*|Θ), where Θ is the vector of unknown population parameters. Let $W_{j}=\;\text{min}\;{\left\{X_{ij}\right\}}_{i=1}^{n_{j}}$ represent the sample minima and $Y_{j}=\;\text{max}\;{\left\{X_{ij}\right\}}_{i=1}^{n_{j}}$ represent the sample maxima, for *j* = 1, …, *m*.

When the sample sizes *n*_*j*_ are known, then the likelihood function is equal to

$$\begin{array}{c} L\left(\Theta \right)=L\left(\Theta |y_{1},\ldots ,y_{m}\right)=\prod_{j=1}^{m}n_{j}F{\left(y_{j}|\Theta \right)}^{n_{j}-1}f\left(y_{j}|\Theta \right) \end{array}$$ (17)

when using the sample maxima, and it is equal to

$$\begin{array}{c} L\left(\Theta \right)=L\left(\Theta |w_{1},\ldots ,w_{m}\right)=\prod_{j=1}^{m}n_{j}{\left(1-F\left(w_{j}|\Theta \right)\right)}^{n_{j}-1}f\left(w_{j}|\Theta \right) \end{array}$$ (18)

when using the sample minima.

When the sample sizes *n*_*j*_ are not known values, but rather are random variables drawn iid from a population with probability mass function *p*(*n*), then the likelihood function is equal to

$$\begin{array}{c} L\left(\Theta \right)=L\left(\Theta |y_{1},\ldots ,y_{m}\right)=\prod_{j=1}^{m}\sum_{\text{all}\;\text{possible}\;n_{j}}n_{j}F{\left(y_{j}|\Theta \right)}^{n_{j}-1}f\left(y_{j}|\Theta \right)p\left(n_{j}\right) \end{array}$$ (19)

when using the sample maxima, and it is equal to

$$\begin{array}{c} L\left(\Theta \right)=L\left(\Theta |w_{1},\ldots ,w_{m}\right)=\prod_{j=1}^{m}\sum_{\text{all}\;\text{possible}\;n_{j}}n_{j}{\left(1-F\left(w_{j}|\Theta \right)\right)}^{n_{j}-1}f\left(w_{j}|\Theta \right)p\left(n_{j}\right) \end{array}$$ (20)

when using the sample minima.

Maximum likelihood estimation chooses to estimate Θ with the value that maximizes the likelihood function *L*(Θ). This value of Θ, called the maximum likelihood estimator or MLE, is often found by differentiating the logarithm of the likelihood function and setting the derivative equal to zero. However, in many cases the equation cannot be solved exactly, and numerical methods must be used in order to approximate the maximum likelihood estimator.

In [7], data from high-voltage power lines was collected to estimate the variance of the corona noise (often heard as a crackling or hissing sound in power lines). Due to limitations of the instruments, only the maximum value of the corona noise was recorded in each three second time lapse, but each maximum value was computed over a sample of size exactly 400. An explicit formula for the maximum likelihood estimator was derived in [7] for the framework of data consisting of sample maxima with equal sample sizes. However, their formula is dependent upon their specific assumption for the population distribution of the corona noise in power lines.

When the population distribution of the *X*_*ij*_ is exponential, as considered in Section 2, then the maximum likelihood estimator can be computed explicitly when using sample minima with known sample sizes, and it is equivalent to our estimator given in Eq (3) when the sample sizes are all equal and is equivalent to our estimator given in Eq (7) when the sample sizes are unequal. When the population is exponentially distributed with known sample sizes, but the data consists of sample maxima, the maximum likelihood estimator cannot be solved for explicitly due to the complexity of the likelihood function. Likewise, when the population is normally distributed or when the sample sizes are unknown with some probability distribution, explicit formulas cannot be found for the maximum likelihood estimators. However, numerical methods can be used to approximate the maximum likelihood estimators in these cases.

Table 1 compares the estimates for the mean *β* of the exponential distribution when using our formula described in Eq (10) versus when approximating the maximum likelihood estimator using the built-in mle function in Matlab for a custom likelihood function of the form given in Eq (19). Likewise, Table 2 compares the estimates for the mean *μ* and variance *σ*^2^ of the normal distribution when using our formulas described in Eq (17) versus when using maximun likelihood estimation. In all cases, 100 simulations were run, each with *m* = 10 sample maxima and with the raw data drawn from either an exponential distribution with true *β* = 3 or a normal distribution with true *μ* = 0 and *σ*^2^ = 1. The *n*_*j*_ values were simulated from five possible probability distributions, all with *E*[*n*_*j*_] set equal to 100. The tables display the mean and standard deviation of the parameter estimates across the 100 simulations for each case, as well as the mean square error (MSE). The mean square error is calculated as the bias (mean of the estimates minus the true parameter value) squared plus the variance of the estimates. Changing the values of *β*, *μ*, *σ*^2^, *m*, and *E*[*n*_*j*_] shifts and rescales the results, but does not change the relative performance of the estimates from our formulas compared to maximum likelihood estimation.

**Table 1:** Comparison of the estimates for the mean of the exponential distribution using Eq (9) versus maximum likelihood estimation, from 100 simulations with true value of *β* = 3.

	$\hat{\beta }$ using Eq (9)			$\hat{\beta }$ using MLE		
Distribution of *n*_*j*_	mean	sd	MSE	mean	sd	MSE
Constant	2.999	0.252	0.064	3.313	0.250	0.161
Uniform, width 10	2.976	0.232	0.054	3.296	0.235	0.143
Uniform, width 50	3.013	0.265	0.071	3.351	0.269	0.195
Uniform, width 100	3.031	0.274	0.076	3.415	0.301	0.263
Poisson	2.996	0.254	0.065	3.331	0.251	0.173

**Table 2:** Comparison of the estimates for the mean and variance of the normal distribution using Eq (15) versus maximum likelihood estimation, from 100 simulations with true value of *μ* = 0 and *σ*^2^ = 1.

	$\hat{\mu }$ using Eq (15)			$\hat{\mu }$ using MLE		
Distribution of *n*_*j*_	mean	sd	MSE	mean	sd	MSE
Constant	-0.012	0.554	0.308	0.141	0.499	0.269
Uniform, width 10	0.034	0.556	0.310	0.149	0.485	0.257
Uniform, width 50	-0.034	0.598	0.358	0.123	0.516	0.281
Uniform, width 100	-0.050	0.675	0.458	0.162	0.568	0.349
Poisson	0.069	0.535	0.291	0.195	0.481	0.269
	${\hat{\sigma }}^{2}$ using Eq (15)			${\hat{\sigma }}^{2}$ using MLE		
Distribution of *n*_*j*_	mean	sd	MSE	mean	sd	MSE
Constant	1.077	0.491	0.247	0.946	0.433	0.190
Uniform, width 10	1.029	0.495	0.246	0.926	0.397	0.163
Uniform, width 50	1.080	0.542	0.300	0.945	0.415	0.175
Uniform, width 100	1.119	0.667	0.459	0.944	0.509	0.262
Poisson	0.993	0.479	0.229	0.890	0.414	0.184

The results displayed in Tables 1 and 2 indicate that the parameter estimates from our formulas tend to be centered closer to the true values on average, whereas maximum likelihood estimation consistently overestimates *β* and *μ*, while consistently underestimating *σ*^2^. When the population is exponentially distributed, our formulas produce similar variability in the parameter estimates and a substantially smaller mean square error compared to maximum likelihood estimation. When the population is normally distributed, maximum likelihood estimation produces smaller variability in the parameter estimates and a slightly smaller mean square error compared to our formulas. However, the fact that our formulas almost always result in less bias indicates that our approach is a valuable alternative to maximum likelihood estimation in both cases. Moreover, the key advantage of our methodology is that we have presented explicit formulas for estimators that can be computed quickly by simply plugging in sample data.

## 5 Biological application

We return now to the example described in Section 1 regarding estimating the mean pollen tube length based upon measurements of the longest pollen tube length in a sample of plants. In the laboratory experiments performed by Swanson et al., either *m* = 8 or *m* = 9 individual plants were used for each time point (3, 6, 9, or 24 hours) for each accession (Columbia or Landsberg) of *Arabidopsis thaliana*. In [1], the mean pollen tube length was estimated assuming a constant *n* = 933 number of pollen tubes within each plant of the Columbia accession and a constant *n* = 727 number of pollen tubes within each plant of the Landsberg accession. The estimation was performed assuming that the individual pollen tube lengths are exponentially distributed, which was shown to be a reasonable fit for the data.

The number of pollen tubes is not in fact constant across all the plants, with the 933 and 727 values actually representing the average number of pollen tubes within each plant for the Columbia and Landsberg accessions, respectively. Data from [2] indicates that it is reasonable to assume that the number of pollen tubes within each plant follows a uniform distribution, with range (640, 1226) for the Columbia accession and range (342, 1112) for the Landsberg accession. Hence, we apply our estimation framework derived in Section 2.3 to estimate the overall mean pollen tube length taking into account the probability distribution of the sample sizes. The resulting estimates, along with the original estimates assuming a constant sample size, are listed in Table 3.

**Table 3:** The estimated mean pollen tube length in mm, assuming either a constant or uniform random number *n*_*j*_ of pollen tubes within each of the *m* plants.

Accession	time (hrs)	*m*	Estimated Mean (mm) assuming	
			Constant *n*_*j*_	Uniform Random *n*_*j*_
Columbia (*E*[*n*_*j*_] = 933)	3	9	0.0931	0.0933
	6	8	0.1442	0.1445
	9	9	0.3423	0.3431
	24	9	0.3746	0.3755
Landsberg (*E*[*n*_*j*_] = 727)	3	9	0.0661	0.0666
	6	8	0.0944	0.0950
	9	8	0.2505	0.2523
	24	9	0.3245	0.3268

The original estimates assuming a constant sample size *n* are equivalent to the estimates that arise when the sample sizes are random and we simply use the average sample size *E*[*n*_*j*_] in place of *n*. However, it was shown in Section 2.4 that simply replacing the constant sample size with the mean sample size leads to a biased estimator that tends to underestimate the true population mean. Thus, although the two sets of estimates are fairly similar, the new estimates, which take into account the distribution of the sample sizes, are more likely to be closer to the true mean pollen tube lengths.
